# Body Mass Index and Risk of Age-Related Cataract: A Meta-Analysis of Prospective Cohort Studies

**DOI:** 10.1371/journal.pone.0089923

**Published:** 2014-02-24

**Authors:** Juan Ye, Li-Xia Lou, Jin-Jing He, Yu-Feng Xu

**Affiliations:** Department of Ophthalmology, the Second Affiliated Hospital of Zhejiang University, College of Medicine, Hangzhou, Zhejiang, China; Case Western Reserve University, United States of America

## Abstract

**Background:**

Age-related cataract (ARC) is the leading cause of blindness in the world. The relationship between body mass index (BMI) and risk of ARC is controversial across observational studies. We therefore performed this meta-analysis to evaluate the association between BMI and risk of ARC.

**Methods:**

Eligible studies were identified through an electronic search of PubMed, Embase and the Cochrane Library. We pooled study-specific relative risks (RRs) and 95% confidence intervals (CIs) to determine the risk of ARC associated with BMI categories and per 1 kg/m^2^ increase in BMI.

**Results:**

A total of 17 prospective cohort studies were included in the meta-analysis. The pooled RRs of ARC were 1.08 (95% CI, 1.01–1.16) for overweight and 1.19 (95% CI, 1.10–1.28) for obesity compared with normal weight. These findings were robust when stratified by sex, sample source, outcome types and confounders, while significantly differed by assessment of BMI and ARC, and duration of follow-up. The summary RR suggested that per 1 kg/m^2^ increase in BMI was associated with a 2% increased risk of ARC (RR 1.02, 95% CI 1.01–1.03). Pooled estimates of RRs consistently indicated a trend for subjects with a high BMI to develop posterior subcapsular cataracts (RR 1.19, 95% CI 1.06–1.35, for overweight; RR 1.50, 95% CI 1.24–1.81, for obesity; RR 1.04, 95% CI 1.01–1.06, per 1 kg/m^2^ increase in BMI) other than nuclear or cortical cataracts.

**Conclusions:**

The overall findings suggest that elevated BMI may increase the risk of ARC, especially posterior subcapsular cataracts. Further trials are needed to investigate the effect of weight reduction in obese populations on the risk of ARC.

## Introduction

Age-related cataract (ARC) is a common ocular disease characterized by lens opacities and visual impairment, which develops slowly as a consequence of aging. Opacities originate in the nucleus, cortex or the posterior pole of the lens, resulting in nuclear, cortical or posterior subcapsular cataracts (PSCs), respectively [1]. Visual impairment and blindness caused by ARC remain major public health problems throughout the world [2]. Although cataract extraction is an effective treatment, many people in developing countries still suffer from cataracts due to high surgery costs and inadequate medical care [3]. The pathogenesis of ARC is multifactorial and not completely understood yet. A number of epidemiologic studies have identified several factors associated with an increased risk of ARC among population worldwide, such as age, smoking, alcohol consumption and ultraviolet radiation [4]–[6].

Compared with normal weight, defined by World Health Organization (WHO) as body mass index (BMI) of 18.5-<25 kg/m^2^, excess body weight (overweight defined as BMI of 25-<30 kg/m^2^, and obesity as BMI of ≥30 kg/m^2^) [7], is recognized as a well-known risk factor for some common diseases, such as diabetes mellitus and cardiovascular diseases [8], [9]. The relationship between BMI and risk of cataract is controversial across observational studies, with reports of positive linear relationship [10]–[12], reduced risk in heavier people [13], [14], and no significant relationship [4], [15], [16]. Prospective data from several large population-based studies demonstrate that obesity was associated with an increased risk of ARC, especially PSC [17], [18]. To our best knowledge, a meta-analysis on the association between obesity and ARC does not exist to date. We have therefore performed a meta-analysis to quantitatively summarize the results of existing prospective cohort studies concerning the effect of BMI on the risk of ARC.

## Materials and Methods

### Searching Strategy

A comprehensive literature search of PubMed (1950 to May 2013), Embase (1900 to May 2013) and the Cochrane Library (up to May 2013) was conducted to identify relevant epidemiological studies. We combined a set of search terms, truncated with wildcard characters if necessary: (“cataract” OR “lens opacity” OR “cataract extraction” OR “cataract surgery”) AND (“body mass index” OR “BMI” OR “obesity” OR “adiposity” OR “overweight” OR “fat” OR “body weight”) with language restricted to English. The titles and abstracts were scanned to exclude any clearly irrelevant studies. The full texts of the remaining articles were read to determine whether they contained information on the topic of interest. We contacted the authors of retrieved articles if additional data were needed. In order to find additional references, we also checked the reference lists of the retrieved publications.

### Study Selection

Two investigators (L.X.L. and J.J.H.) independently evaluated all retrieved studies for possible inclusion. To be included, the study had to meet the following criteria: (1) had a prospective cohort study design; (2) reported BMI categories identical or similar to the WHO recommended classifications of body weight [7]; (For Asian populations, WHO suggests categories as follows: 18.5-<23 kg/m^2^ normal weight; 23-<27.5 kg/m^2^ overweight; and ≥27.5 kg/m^2^ obesity [19].) (3) the outcome measure was incident cataract or cataract extraction; (4) reported effect estimates (relative risks (RRs), odds ratios (ORs) or hazard ratios (HRs)) with corresponding 95% confidence intervals (CIs) for BMI categories associated risk of ARC, provided effect estimates per unit (in kg/m^2^) increase in BMI, or allowed for calculation from raw data contained in the article. If more than one study used the same cohort, the one with the most comprehensive population or longest follow-up time was included. We excluded any article with inconsistent or erroneous data. Conference abstracts or unpublished reports were not considered.

### Data Extraction and Study Quality Assessment

Two investigators (L.X.L. and J.J.H.) independently extracted data from each included study. Any disagreements were resolved through discussion with the senior investigator (J.Y.). We collected the following information, including last name of first author, year of publication, study location, mean follow-up time, sample size, age range of subjects, BMI determinants, case definitions of ARC, outcome types (incident cataract including nuclear cataract, cortical cataract and PSC, or cataract extraction), covariates adjusted in multivariable analysis, and effect estimates for BMI categories or per unit increase in BMI. The BMI categories that were closest to the WHO definition of weight status were applied if non-standard categories were used in individual studies included in this meta-analysis. If a study had several adjustment models, we extracted the one that reflected the maximum extent of adjustment for potentially confounding variables. If no adjusted estimates were available, unadjusted estimates were used. We extracted sex-specific data *i*f they were available in individual studies.

Study quality assessment was performed using the Newcastle-Ottawa scale [20], which is validated for non-randomised studies in a meta-analysis. This scale awards a maximum of nine points to each study: four for selection of participants and measurement of exposure (BMI); two for comparability of cohorts on the basis of the design or analysis; and three for assessment of outcomes (incident cataract or cataract extraction) and adequacy of follow-up.

### Statistical Analysis

The RRs with corresponding 95% CIs were considered as the effect estimates for all included studies. Any results stratified by sex were treated as two separate reports. If a study only reported results specific for ARC subtypes, the subtype-specific RRs were pooled as an estimate for ARC of any type. Study-specific RRs were pooled using fixed-effects models with the Mantel-Haenszel method when heterogeneity was negligible, or random-effects models with the DerSimonian-Laird method when heterogeneity was significant [21]. We evaluated the risk of ARC in overweight and obese subjects in contrast to normal weight subjects. Also, we analysed the linear relationship between BMI and risk of ARC. Several studies included in this meta-analysis reported RRs for cataracts associated with per 1 kg/m^2^ increase or per 5 kg/m^2^ increase in BMI. If the RR was on the 5 kg/m^2^ increase scale, the log RR and its standard error were divided by 5 to derive the corresponding 1 kg/m^2^ increase estimate [22].

Statistical heterogeneity among studies was evaluated using the Cochran’s *Q* test and *I^2^* statistic [23]. Heterogeneity was confirmed with a significance level of *P*<0.10. *I^2^* values of <25%, 25%-<50%, 50%-<75% and ≥75% were considered as no, low, moderate and high heterogeneity, respectively. Sensitivity analysis was performed to investigate the contribution of each study to the heterogeneity by sequentially removing one study and reanalyzing the pooled estimate for the remaining studies. We also conducted subgroup analyses investigating whether sex, sample source, BMI and ARC ascertainments, duration of follow-up, outcome types, ARC subtypes, and adjustment for confounders influenced the results [24]. Publication bias assessment was done using the Egger regression asymmetry test and the Begg adjusted rank correlation test [25], [26]. All statistical analyses were performed with Stata 12.0 (Stata Corporation, College Station, TX). For all analyses, except heterogeneity, *a value of P*<0.05 was regarded as statistically significant, and all tests were two-sided.

## Results

### Literature Search and Characteristics of Included Studies

**Figure 1:**
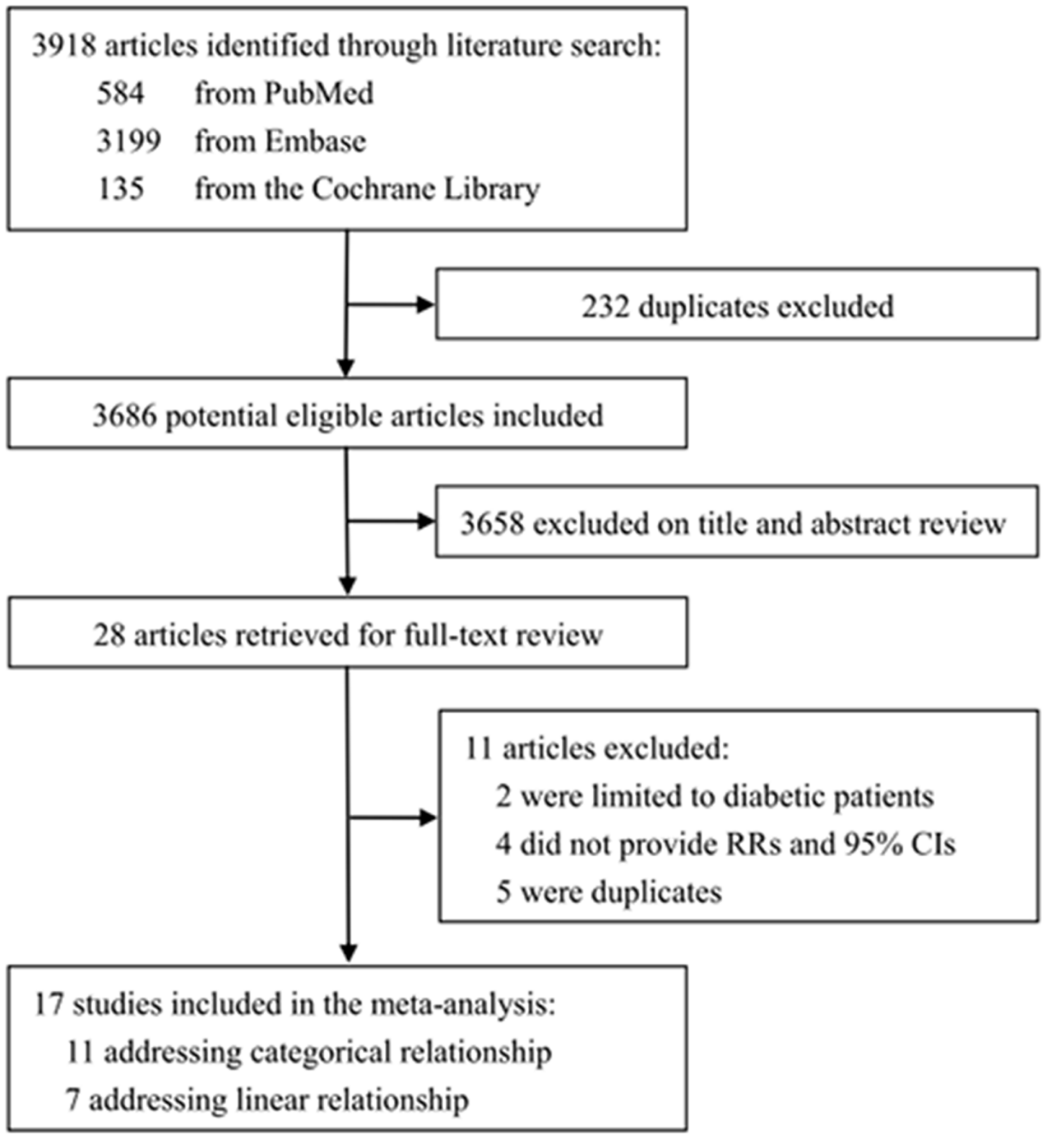
Flow diagram of study selection process.

The literature search yielded 4340 articles from the electronic database. After removing 391 duplicate publications, 3949 studies were considered of potential relevance (Fig. 1). In total, 28 articles were retrieved for full-text review, 19 of which met all the predefined inclusion criteria. However, we excluded studies that were limited to non-generalizable patients, including 2 studies of ARC in diabetic patients [27], [28]. Finally, 17 prospective cohort studies were included in this meta-analysis. 11 studies were eligible for the categorical relationship between BMI and ARC [4], [12], [15]–[18], [29]–[33], 1 of which only evaluated nuclear cataract [18]. 7 studies were eligible for the linear relationship [10]–[13], [34]–[36], 2 of which only reported incident nuclear cataract [13], [34].

Characteristics of the included studies are summarized in Table 1. ARC definitions and assessment varied among the studies. Diagnosis of cataract or cataract surgery was ascertained by standardized criteria in 9 studies [4], [13], [16]–[18], [31], [34]–[36], and self-reported by questionnaire or interview in 6 studies [10]–[12], [30], [32], [33], while in 2 studies cases were determined based on a national register [15], [29]. 15 studies reported incident cataract as the measure of outcome and 5 studies evaluated cataract extraction. For studies which reported both of the outcome measures [4], [17], [18], [32], we considered incident cataract as the primary outcome. BMI was calculated by measured weight and height in 7 studies [4], [13], [16], [17], [31], [34], [36], and identified by self-reports of anthropometric measurements in 10 studies [10]–[12], [15], [18], [29], [30], [32], [33], [35]. Results of quality assessment yielded a score range from 5 to 9 for the included studies with an average score of 6.7. The influences of the indicators of study quality on the relationship between BMI and ARC, such as sample source, exposure and outcome determinants, duration of follow-up, and adjustment for confounders, were then investigated in subgroup analyses.

**Table 1:** Characteristics of Studies Included in the Meta-Analysis.

Authors (published year, location)	Years offollow-up	Participants	Age range, y	Sample size	BMI determinants	ARC definitions	Outcome types	Study quality
Schaumberg et al.^32^ (2000, USA)	13.7	Volunteers	40–84	20271 men	Self-reported	Self-reported with confirmation	Any type, NC, CC, PSC, Extraction	6
Tan et al.^17^ (2008, Australia)	10.5	Population-based	≥49	2564	Measured	Standard criteria	NC, CC,PSC, Extraction	9
Weintraub et al.^12^ (2002, USA)	10 (male) 16 (female)	Volunteers	≥45	45549 men 87682 women	Self-reported	Self-reported with confirmation	Extraction (Any type, NC,PSC)	6
Yoshida et al.^33^ (2000, Japan)	5	Population-based	≥50	35365 men 40825 women	Self-reported	Self-reported	Any type	6
Lindblad et al.^15^ (2008, Sweden)	8.2	Population-based	49–83	35369 women	Self-reported	National register	Extraction	6
Hiller et al.^31^ (1998, USA)	12.5	Population-based	52–80	714	Measured	Standard criteria	Any type, NC, CC, PSC	8
Richter et al.^16^ (2012, USA)	4	Population-based	≥40	3471	Measured	Standard criteria	NC, CC, PSC	6
Chang et al.^4^ (2011, USA)	9.8	Hospital-based	55–80	4425	Measured	Standard criteria	Any type, NC, CC, PSC, Extraction	7
Appleby et al.^29^ (2011, UK)	11.4	Population-based	≥40	27670	Self-reported	National register	Any type	7
Chodick et al.^30^ (2008, USA)	20	Volunteers	23–44	35705	Self-reported	Self-reported	Any type	6
Mares et al.^18^ (2010, USA)	6	Population-based	50–79	1808 women	Self-reported	Standard criteria	NC	7
Glynn et al.^10^ (2009, USA)	13	Volunteers	40–84	20599 men	Self-reported	Self-reported with confirmation	NC, CC, PSC	6
Klein et al.^35^ (2003, USA)	5	Population-based	43–84	4926	Self-reported	Standard criteria	NC, CC, PSC	7
Williams.^11^ (2009, USA)	7	Volunteers	38–44	29025 men 11967 women	Self-reported	Self-reported	Any type	5
Zhang et al.^36^ (2011, China)	5	Population-based	≥45	3251	Measured	Standard criteria	NC, CC, PSC	6
Leske et al.^13^ (2002, Barbados)	4	Population-based	40–84	3427	Measured	Standard criteria	NC	8
Karppi et al.^34^ (2012, Finland)	4	Population-based	61–80	1689	Measured	Standard criteria	NC	8

ARC: age-related cataract; BMI: body mass index; CC: cortical cataract; NC: nuclear cataract; PSC: posterior subcapsular cataract.

### The Association between BMI Categories and Risk of ARC

**Figure 2:**
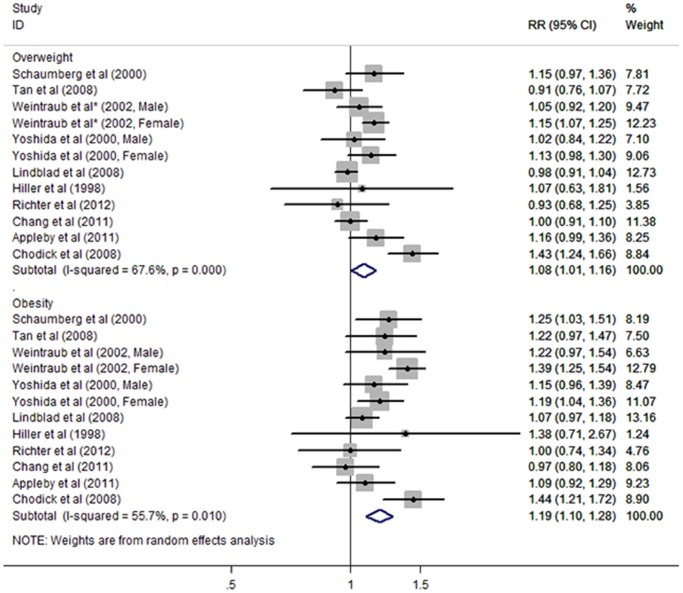
Forest plot of risk estimates of age-related cataract associated with overweight and obesity. *Derived by combining BMI categories of 25–<27.9 kg/m^2^ and 28-<29.9 kg/m^2^.

The pooled results of 10 studies showed that overweight subjects had a statistically significantly increased risk of ARC (RR 1.08, 95% CI 1.01–1.16; *I^2^* = 67.6%, *P*_heterogeneity_ <0.001) when compared to normal weight subjects (Fig. 2). Sensitivity analyses indicated that the pooled RR was not excessively changed by any individual studies, ranging from 1.05 (95% CI 1.00–1.11) to 1.10 (95% CI 1.02–1.18). Table S1 shows the results of sensitivity analyses. Egger’s and Begg’s test confirmed the absence of publication bias (Egger’s test = 0.614, Begg’s test = 0.945). Obese subjects also had a higher risk of ARC (RR 1.19, 95% CI 1.10–1.28; *I^2^* = 55.7%, *P*_heterogeneity_ = 0.010). Omission of individual studies revealed that no single study had a particular influence on the pooled estimate, detected by pooled RR ranging from 1.15 (95% CI 1.07–1.24) to 1.21 (95% CI 1.12–1.30). No publication bias was detected by Egger’s and Begg’s test (Egger’s test = 0.823, Begg’s test = 1.000).

We performed subgroup analyses to investigate the effect of sex, sample source, BMI and ARC ascertainments, duration of follow-up, outcome types, ARC subtypes, and selected confounders (alcohol use, diabetes and hypertension) on the relationship between BMI categories and ARC (Table 2). Similar risk estimates were found in both genders. Likewise, there was no evidence that the pooled RRs differed significantly by sample source, outcome types or adjustment of confounders. However, ascertainments of exposure and outcome were found to significantly modify the association between ARC and overweight, but not obesity. The pooled RR from studies with measured BMI and standard diagnosis of cataract (overweight: RR 0.98, 95% CI 0.90–1.06) was lower than from studies with self-reported BMI and non-standard definition of cataract (overweight: RR 1.12, 95% CI 1.03–1.22). When pooling results from studies with more than 10 years of follow-up, the RRs were significantly higher than from studies with less than 10 years of follow-up (overweight: RR 1.13, 95% CI 1.03–1.25 versus RR 1.00, 95% CI 0.96–1.05; obesity: RR 1.28, 95% CI 1.19–1.39 versus RR 1.09, 95% CI 1.02–1.17). There was no evidence for significant relationship between BMI categories and cortical or nuclear cataract. However, BMI was strongly associated with PSC (overweight: RR 1.19, 95% CI 1.06–1.35; obesity: RR 1.50, 95% CI 1.24–1.81).

**Table 2:** Subgroup Analyses of Body Mass Index (BMI) Categories and Risk of Age-Related Cataract (ARC).

		Overweight			Obesity		
Subgroup	N ofstudies	Pooled RR(95% CI)	*P*_heterogeneity_	*P*_difference_	Pooled RR(95% CI)	*P*_heterogeneity_	*P*_difference_
All	10	1.08 (1.01–1.16)	<0.001		1.19 (1.10–1.28)	0.010	
Gender							
Male	3	1.08 (0.99–1.18)	0.466	Reference	1.22 (1.09–1.36)	0.711	Reference
Female	3	1.08 (0.96–1.21)	0.006	1.000	1.21 (1.03–1.42)	0.002	0.934
Sample source							
Population-based	5	1.02 (0.95–1.10)	0.165	Reference	1.12 (1.05–1.19)	0.701	Reference
Other	5	1.14 (1.02–1.26)	0.003	0.090	1.26 (1.11–1.43)	0.034	0.102
BMI & ARC ascertainments							
Measured & Standard criteria	4	0.98 (0.90–1.06)	0.775	Reference	1.08 (0.94–1.23)	0.352	Reference
Self-reported & Non-standard	6	1.12 (1.03–1.22)	<0.001	0.026	1.22 (1.12–1.33)	0.010	0.134
Duration of follow-up							
≥10 yrs	6	1.13 (1.03–1.25)	0.007	Reference	1.28 (1.19–1.39)	0.246	Reference
<10 yrs	4	1.00 (0.96–1.05)	0.482	0.025	1.09 (1.02–1.17)	0.433	0.002
Outcome type							
Incident cataract	7	1.08 (0.97–1.21)	0.002	Reference	1.19 (1.08–1.30)	0.151	Reference
Cataract extraction	5	1.04 (0.95–1.15)	0.002	0.613	1.16 (0.99–1.34)	<0.001	0.778
ARC subtype							
Nuclear cataract	7	1.00 (0.90–1.11)	0.131	Reference	1.08 (0.94–1.24)	0.066	Reference
Cortical cataract	5	1.04 (0.92–1.18)	0.364	0.637	1.12 (0.93–1.35)	0.246	0.759
PSC	6	1.19 (1.06–1.35)	0.589	0.033	1.50 (1.24–1.81)	0.224	0.006
Adjustment for confounders							
Alcohol use							
Yes	4	1.13 (0.98–1.30)	<0.001	Reference	1.20 (1.08–1.33)	0.059	Reference
No	6	1.05 (0.98–1.13)	0.102	0.363	1.17 (1.03–1.32)	0.021	0.759
Diabetes							
Yes	5	1.09 (0.94–1.25)	0.001	Reference	1.19 (1.07–1.33)	0.104	Reference
No	5	1.07 (0.99–1.16)	0.030	0.824	1.18 (1.05–1.32)	0.008	0.917
Hypertension							
Yes	3	1.11 (0.92–1.35)	0.001	Reference	1.24 (1.13–1.37)	0.284	Reference
No	7	1.06 (0.99–1.13)	0.058	0.656	1.15 (1.03–1.28)	0.006	0.309

CI: confidence interval; PSC: posterior subcapsular cataract; RR: relative risk.

### The Association between per 1 kg/m^2^ Increase in BMI and Risk of ARC

**Figure 3:**
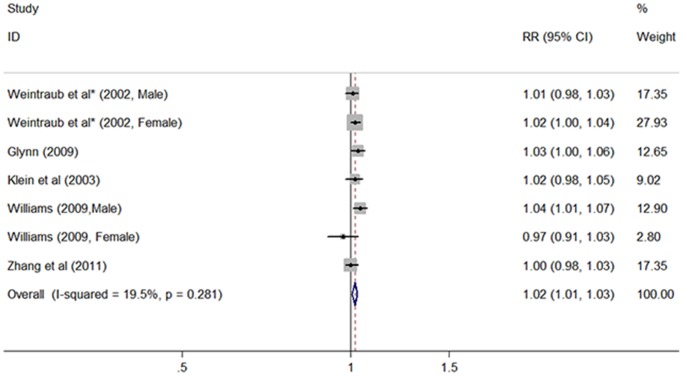
Forest plot of risk estimates of age-related cataract associated with per 1 kg/m^2^ increase in BMI. *Derived from RRs associated with per 5 kg/m^2^ increase in BMI.

We performed fixed-effects meta-analysis of 5 studies to determined the risk of ARC associated with per 1 kg/m^2^ increase in BMI. Figure 3 shows that an increase in BMI of 1 kg/m^2^ was statistically significantly associated with ARC (RR 1.02, 95% CI 1.01–1.03; *I^2^* = 19.5%, *P*_heterogeneity_ = 0.281). Egger’s and Begg’s test confirmed no evidence of publication bias. Stratified analyses by ARC subtypes revealed differences among the three subtypes (RR 1.00, 95% CI 0.98–1.02, for nuclear cataract, 6 cohorts; RR 1.01, 95% CI 0.99–1.02, for cortical cataract, 3 cohorts; RR 1.04, 95% CI 1.01–1.06, for PSC, 4 cohorts).

## Discussion

This meta-analysis of 11 prospective cohort studies addressing the categorical relationship between BMI and ARC revealed that overweight and obesity were significantly associated with an increased risk of ARC, especially PSC. This positive relationship is independent of sex, sample source, outcome types, and selected confounders, such as alcohol intake, diabetes and hypertension, while significantly differed by assessment of BMI and ARC, and duration of follow-up. However, one must be wary of generalizing results from subgroup analyses because of relatively fewer studies involved.

There was evidence of moderate heterogeneity among the studies included in the categorical analysis. Adjustment of confounders was different among the studies and residual confounding may have affected the individual effect estimates and thus the pooled RR. All these studies, except the study by Richter et al [16], evaluated the relationship between BMI and ARC after adjusting for potential confounders, such as age and smoking. When removing the study by Richter et al [16], the risk estimates were essentially unchanged (Table S1).A wide variation existed among the cohorts with regard to the duration of follow-up. Studies with more than 10 years of follow-up showed a stronger positive association between BMI and risk of ARC than studies with shorter follow-up duration (Table 2). Considering that cataracts progress at various rates in different individuals to the point where they are detected and require extraction, achieving adequate follow-up in cohorts is essential to establish the validity of the findings. We noticed that a large proportion of the studies were conducted in non-Asian regions, in keeping with the high prevalence of obesity in these regions and associated research efforts. After exclusion of the only study conducted in Asia, the summary RR remained stable (Table S1) [33].

We also found a linear positive association between BMI and risk of ARC. Per 1 kg/m^2^ increase in BMI was associated with a 2% increased risk of ARC, with no heterogeneity detected (*I^2^* = 19.5%). Caution must be taken in the interpretation of this pooled estimate. Although the pooled effect size represents only a weak positive association, its interpretation depends on the unit of measurement. A 5 kg/m^2^ increase in BMI equates to a much stronger association (RR 1.10). Similarly, subgroup analyses found that per 1 kg/m^2^ increase in BMI was more strongly associated with the risk of PSC than other types of ARC, as well as the results of BMI categorical analysis. Likewise, we cannot exclude type I error, because the sample size was small in these subgroups.

Strengths of our meta-analysis included the quantitative analyses based on prospective cohort studies, which tend to be less susceptible to recall and selection bias than retrospective case-control studies. Moreover, most cohorts were population-based with findings being more generalizable. The large number of included studies allowed us to investigate both the categorical and linear relationship between BMI and ARC, and to better explore the effect of excess weight on various subgroups.

However, our meta-analysis has several limitations that may affect the interpretation of the results. First, the included studies exhibited wide differences in outcome definition. Because several studies relied on participants to self-report their diagnoses of cataract, underestimates of the number of cases might have occurred due to a few false-negative cases. Besides, cataract surgery depends on health services provision and access, which would produce bias if different between normal weight and excess weight individuals. Second, in some studies BMI determinants were dependent on self-reporting questionaires. A downward bias in self-reported BMI especially of heavier individuals has been found to result in misclassification of weight status [37]. However, a few included studies had validated self-reported information with correlation of self-reported weight versus measured weight ranging from 0.96 to 0.97 [12], [32]. Third, as in any meta-analysis, the possibility of publication bias is of concern because studies which have results that are not statistically significant or have been previously published may be less likely to be published. However, statistical tests did not provide evidence of publication bias in our meta-analysis.

There are several plausible pathophysiological pathways through which increased BMI might promote cataract formation. First, obese individuals have elevated plasma levels of leptin [38], which might be involved in lens opacity for enhancing accumulation of reactive oxygen species [39]–[41]. Second, individuals with obesity have more intense systemic inflammation with elevated levels of C-reactive protein and pro-inflammatory cytokines [42], both of which could promote the development of cataract [43], [44]. Third, obesity might be linked to cataract by its complications such as diabetes, hyperlipidemia and hypertension [8], [38], [45], which are all known risk factors for cataract [46], [47]. The stronger association between BMI and PSC might be due to the different patterns of formation and progression of ARC subtypes. Although less common than nuclear and cortical opacities, PSCs are more likely to result in visual disability, and are highly overrepresented among extracted cataracts [41].

In summary, findings from our meta-analysis indicate that high BMI may increase the risk of ARC in both sexes, especially PSC. As obesity prevalence continues to be on an upward trajectory worldwide [48], the contribution of obesity to the development of ARC might constitute a proportion of the global burden of ARC. The results of our meta-analysis imply that beneficial lifestyle changes to reduce body weight, which clearly have other health benefits as well, might help to lessen the incidence and associated costs of cataract. In future research, randomized trials are needed to examine the effect of weight reduction in obese populations on the risk of ARC.

## Supporting Information

Checklist S1 PRISMA checklist.
(DOC)

Table S1 Results of Leave-One-Out Sensitivity Analyses.
(DOCX)
