# Supplementary material for: Body Mass Index and Risk of Age-Related Cataract: A Meta-Analysis of Prospective Cohort Studies
Source: PLoS One. 2014 Feb 24;9(2):e89923. doi: 10.1371/journal.pone.0089923 (PMC3933700; doi:10.1371/journal.pone.0089923)
Supplement: Table S1 — Results of Leave-One-Out Sensitivity Analyses. (DOCX) [file pone.0089923.s002.docx]

**Table S1.** Results of Leave-One-Out Sensitivity Analyses

|  | Overweight | |  | Obesity | |
| --- | --- | --- | --- | --- | --- |
| Study Excluded | Pooled RR (95% CI) | *P*_heterogeneity_ |  | Pooled RR (95% CI) | *P*_heterogeneity_ |
| Schaumberg et al^32^ | 1.07 (1.00-1.16) | <0.001 |  | 1.18 (1.09-1.28) | 0.006 |
| Tan et al^17^ | 1.10 (1.02-1.18) | 0.001 |  | 1.18 (1.09-1.28) | 0.006 |
| Weintraub et al^12^ | 1.07 (0.99-1.17) | 0.001 |  | 1.15 (1.07-1.24) | 0.120 |
| Yoshida et al^33^ | 1.08 (1.00-1.17) | <0.001 |  | 1.19 (1.08-1.31) | 0.003 |
| Lindblad et al^15^ | 1.10 (1.02-1.18) | 0.005 |  | 1.21 (1.12-1.30) | 0.043 |
| Hiller et al^31^ | 1.08 (1.01-1.16) | <0.001 |  | 1.18 (1.09-1.28) | 0.006 |
| Richter et al^16^ | 1.09 (1.01-1.17) | <0.001 |  | 1.20 (1.11-1.29) | 0.009 |
| Chang et al^4^ | 1.09 (1.01-1.18) | <0.001 |  | 1.21 (1.12-1.30) | 0.027 |
| Appleby et al^29^ | 1.07 (1.00-1.16) | <0.001 |  | 1.20 (1.10-1.30) | 0.009 |
| Chodick et al^30^ | 1.05 (1.00-1.11) | 0.066 |  | 1.16 (1.08-1.26) | 0.029 |

CI: confidence interval; RR: relative risk.
